# Evaluation of different platforms for the detection of anti-SARS coronavirus-2 antibodies, Thailand

**DOI:** 10.1186/s12879-021-06921-y

**Published:** 2021-12-06

**Authors:** Hatairat Lerdsamran, Anek Mungaomklang, Sopon Iamsirithaworn, Jarunee Prasertsopon, Kriengkrai Prasert, Poj Intalapaporn, Nirada Siriyakorn, Witthawat Wiriyarat, Nattakan Thinpan, Suteema Sawadpongpan, Somrak Sirikhetkon, Noparat Mongkalangoon, Suwanna Petto, Pilaipan Puthavathana

**Affiliations:** 1 Center for Research and Innovation, Faculty of Medical Technology, Mahidol University, Nakhon Pathom, 73170 Thailand; 2 Institute for Urban Disease Control and Prevention, Department of Disease Control, Ministry of Public Health, Bangkok, 10220 Thailand; 3 Department of Disease Control, Ministry of Public Health, Nonthaburi, 11000 Thailand; 4 Department of Medical Services, Nakhon Phanom Provincial Hospital, Ministry of Public Health, Nakhon Phanom, 48000 Thailand; 5 Division of Infectious Disease, Department of Medicine, Rajavithi Hospital, Ministry of Public Health, Bangkok, 10400 Thailand; 6 Faculty of Veterinary Science, Mahidol University, Nakhon Pathom, 73170 Thailand

**Keywords:** SARS-coronavirus-2, Antibody detection, Microneutralization assay, ELISA, Chemiluminescence assay

## Abstract

**Background:**

Antibodies against severe acute respiratory syndrome coronavirus 2 (SARS-CoV-2) help determine previous infection in individuals, regardless of whether they are asymptomatic or symptomatic. The detection of antibodies serves several purposes, including supporting other assays for disease diagnosis, conducting seroepidemiological studies, and evaluating vaccines. Many platforms of immunological methods for anti-SARS-CoV-2 antibody detection and their performance require validation.

**Methods:**

This study evaluated the test performance of three autoanalyzer-based assays (Architect IgG, Vitros IgG, and Vitros total Ig) and one manual ELISA (Wantai total Ig) against a microneutralization (microNT) assay on the detection of SARS-CoV-2 antibodies. Furthermore, an indirect immunofluorescence assay verified the discordant results between the microNT and commercial assays. The test sensitivity, specificity, positive predictive value, and negative predictive value were determined based on four groups of 1005 serum samples: 102 COVID-19 prepandemic sera, 45 anti-SARS-CoV-2 positive sera, 366 sera of people at risk, and 492 sera of citizens returning from countries with a high prevalence of infection.

**Results:**

The analyses as a whole showed that the performance of these commercial assays was comparable. Each group was also analysed separately to gain further insight into test performance. The Architect did not detect two positive sera of people at risk (prevalence of infection 0.55%). The other methods correctly identified these two positive sera but yielded varying false-positive results. The group of returning travellers with an infection rate of 28.3% (139 of 492) better differentiated the test performance of individual assays.

**Conclusions:**

High-throughput Architect and Vitros autoanalyzers appear appropriate for working on large sample sizes in countries that can afford the cost. The Wantai ELISA, while requiring more individual time and technical skill, may provide reliable results at a lower cost. The selection of assays will depend on the laboratory facilities and feasibility.

## Background

Almost all immunocompetent individuals infected with severe acute respiratory syndrome coronavirus 2 (SARS-CoV-2) develop antibodies specific to multiple viral proteins. In particular, antibodies to nucleoprotein (N) and spike (S1 and S2) proteins are of clinical importance [1–4]. Specific IgM and IgA first appeared 7–14 days after the onset of disease symptoms, followed by IgG at approximately 14 days. IgM peaks at 2–5 weeks and then declines a few weeks later, while IgG may persist longer [3, 5–8]. Anti-N antibodies developed before the anti-S antibodies [9, 10]. Various immunological methods have demonstrated the binding activities of these immunoglobulin (Ig) isotypes, e.g., enzyme-linked immunosorbent assay (ELISA), chemiluminescence immunological assays (CLIAs), indirect immunofluorescence (IIF) assay, and immunochromatography. Additionally, plaque reduction neutralization (PRNT) or microNT assays detected functional or neutralizing (NT) antibodies. NT antibodies correlate with protective immunity, while binding antibodies may or may not [3, 8, 10–12]. Antibodies to the receptor-binding domain (RBD) in the S1 protein correlated well with NT antibody activity [2, 3, 10, 12].

Antibody detection has served many purposes: supporting the diagnosis of SARS-CoV-2 infection when reverse transcription-polymerase chain reaction (RT-PCR) for viral genomes yields an inconclusive result [3]; serosurveillance to estimate the cumulative incidence of SARS-CoV-2 infection [3, 13–15]; and vaccine evaluation, particularly by PRNT or microNT assays.

Currently, multiple commercial kits are available globally, and antigenic targets for these tests include the SARS-CoV-2 S, RBD, and N proteins [1–3]. Evaluations of most serological test kits used sera from RT-PCR-confirmed cases as the gold standard for comparison with COVID-19 prepandemic sera [16] or RT-PCR-negative sera [17]. Few studies have included assessments of SARS-CoV-2 binding antibodies against functional NT antibodies [8, 18]. Many of these test kits require autoanalyzer machines that are expensive and inaccessible to laboratories in developing countries. Manual ELISAs of comparable performance may have broad utility in lower resource settings.

Therefore, we evaluated four serological assays that used different platforms against the microNT assay as the gold standard method for the detection of anti-SARS-CoV-2 antibodies. The evaluation included two autoanalyzers using three chemiluminescence-based kits (Architech IgG, Vitros IgG, and Vitros total Ig) and a manual ELISA for total Ig (Beijing Wantai). We retested all samples with IIF to validate the discordant results of the microNT and the evaluated test kits. The test sera in this study included COVID-19 prepandemic sera, NT antibody-positive sera from SARS-CoV-2 infected cases, sera of persons at risk of SARS-CoV-2 infection, and sera of travellers returning from countries experiencing SARS-CoV-2 outbreaks at the time of this study.

## Methods

### Ethical issues and the test sera

This study was conducted following the Declaration of Helsinki and approved by the Mahidol University Central Institutional Review Board (MU-CIRB). The study comprised 1005 convenience serum samples from four groups of participants: (1) Archival COVID-19 prepandemic serum samples collected in 2019 before the emergence of SARS-CoV-2 as the negative controls (N = 102). Participants provided informed consent for using their leftover sera in other research projects under the approval of the MU-CIRB protocol number MU-IRB 2017/180.1210; (2) Anonymized archival sera collected between 10 and 109 days after disease onset from cases with RT-PCR confirmed SARS-CoV-2 infection in 2020 as the positive controls (N = 45). These sera were kindly provided by Rajvithi Hospital. MU-CIRB waived the requirement for informed consent because the sera were the leftover samples from a clinical laboratory investigation under the Protocol number MU-COVID2020.001/2503; (3) Serum samples collected in 2020 from 366 Thai people determined by the Ministry of Public Health (MoPH) to be at increased risk of infection (health personnel, taxi drivers, and workers in entertainment venues). The informed consent in this group of participants was also provided by the MU-CIRB protocol number MU-COVID2020.001/2503. In the enrolment process, survey staff explained the purpose of the study. Participants signed an informed consent form for the interview about demographics and occupational activities as well as the donation of approximately 5–8 ml of blood using venipuncture. Blood specimens were labelled with study ID numbers. (4) Anonymous serum samples from 492 Thai citizens in state quarantines after returning to Thailand in 2020 from extended duty in countries with a known SARS-CoV-2 outbreak at the time of their return. These sera were sent from the Institute for Urban Disease Control and Prevention (IUDC), Department of Disease Control, MoPH for anti-SARS-CoV-2 antibody testing to support active case surveillance by RT-PCR. The outbreak investigation is considered a public health intervention; therefore, the Ethical Review Committee for Research related to COVID-19 Disease or Public Health Emergency, Department of Disease Control, MoPH (DDC ERC) exempted IRB approval and written informed consent due to its urgent nature. Nevertheless, participants received an explanation and gave verbal informed consent on specimen collection for laboratory diagnosis of SARS-CoV-2 infection. We included the laboratory results in our study analysis with permission from IUDC.

### Study design

We evaluated the Wantai ELISA total Ig, Architect IgG, Vitros IgG, and Vitros total Ig tests against the microNT test as the reference assay. By its nature, microNT is less sensitive than binding antibody assay techniques. Therefore, IIF was used to retest specimens when the microNT and a commercial assay yielded discordant results. IIF may support the result of microNT or that of the commercial assay. The final result of a sample with discordant results followed that of the IIF assay.

### Microneutralization assay

The present study conducted the microNT assay as described previously [19]. Vero cells (African green monkey kidney cells—ATCC, CCL-81) were grown in Eagle’s minimum essential medium (EMEM) (Gibco, NY) supplemented with 10% fetal bovine serum (FBS) (Gibco, NY), antibiotics, and amphotericin B. SARS-CoV-2 (hCoV-19/TH/MUMT-4/2020) was isolated and propagated in Vero cell cultures maintained in EMEM supplemented with 2% FBS. We calculated the virus concentration in the 50% tissue culture infective dose (TCID50) using the Reed–Muench method. We performed the experiments using the infectious viruses in a level 3 biosafety laboratory.

The cytopathic effect (CPE)-based microNT assay employed SARS-CoV-2 at a final concentration of 100 TCID50/100 µl and Vero cells grown in 96-well microculture plates in duplicate. The test serum was heat-inactivated at 56 °C for 30 min and serially diluted twofold starting from an initial serum dilution of 1:10 up to 1:1280. Then, 60 µl of each serum dilution was mixed with 60 µl of the test virus (200 TCID50/100 µl). After incubation at 37 °C for 1 h, 100 µl of the virus serum mixture was added into each well containing the Vero cell monolayer. The reaction plates were further incubated at 37 °C and observed daily for 3 days before the results were obtained. The neutralizing antibody titer was the highest reciprocal serum dilution that inhibited ≥ 50% CPE of the cell monolayer inoculated with the serum–virus mixture compared to the uninfected cultures.

### Indirect immunofluorescence assay

Vero cell monolayers were infected with SARS-CoV-2 for 3–4 days and harvested when the infected cultures showed 50–75% CPE. The cells were scraped off the surface of the culture flask, suspended, washed, and spun. Then, the cell pellets were suspended, spotted onto microscopic glass slides, and air-dried before fixing in precooled acetone at − 20 °C for 10 min. The cell deposits were applied with rabbit monoclonal antibodies to SARS-CoV-2 N or spike S1 protein (Sino Biological, Beijing, China) and incubated at 37 °C for 60 min. After washing, goat anti-rabbit IgG (H + L)-conjugated Alexa Fluor^®^ 488 (Invitrogen, Waltham, MA USA) was added, followed by the same staining procedure. The standard cell deposit contained approximately 50–75% antigen-positive cells.

**Fig. 1:**
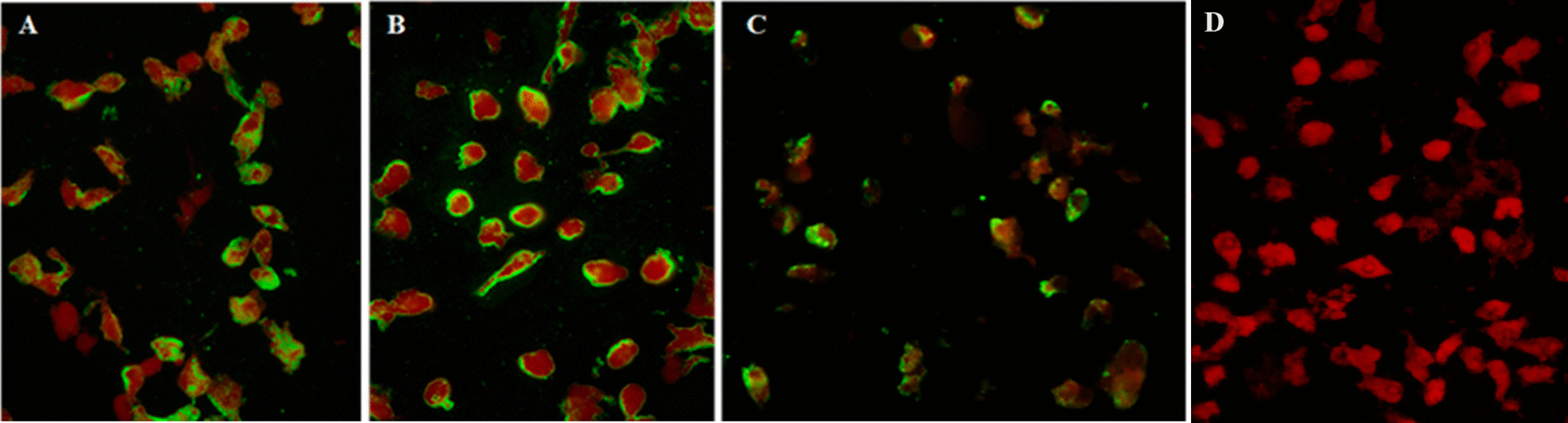
Indirect immunofluorescence (IIF) assay of SARS-CoV-2-infected Vero cells stained with **A** monoclonal antibody to S epitopes; **B** monoclonal antibody to N epitopes; and **C** human serum at a dilution of 1:10. This serum sample was negative by microNT but positive by Wantai ELISA, Vitros total Ig, and IIF; and **D** human negative serum control. The fluorescent positive cells appeared apple green in the cytoplasm of the infected cells

The staining for anti-SARS-CoV-2 antibodies in human sera employed a single serum dilution of 1:10 in a phosphate-buffered saline, and polyclonal rabbit anti-human IgA, IgG, IgM, Kappa, and Lambda conjugated with fluorescein isothiocyanate (Dako, Glostrup, Denmark) in Evans blue diluent were used as the secondary antibodies. The viral antigen localized in the cytoplasm of the infected cells appeared fluorescent apple green under a fluorescence microscope (Fig. 1).

### Wantai ELISA for SARS-CoV-2 total Ig

Sandwich ELISA (Wantai Biological Pharmacy, Beijing, China) for detecting anti-SARS-CoV-2 total antibodies was determined using a plate precoated with the RBD of the SARS-CoV-2 spike protein. The reaction consisted of two steps. First, 100 µl of the serum was incubated with the RBD at 37 °C for 30 min before washing. Second, horseradish peroxidase (HRP)-conjugated RBD was added to each reaction well and further incubated at 37 °C for 30 min before washing and adding a chromogenic solution for 15 min. The amount of the SARS-CoV-2 antibodies was proportional to the optical density values as measured under a spectrophotometer using dual wavelengths of 450 nm and 630 nm. The absorbance value (A) of the test sample was divided by the cut-off value (C.O.) to determine the A/C.O. ratio. Ratios of ≥ 1 were considered positive for SARS-CoV-2 antibodies, and ratios between 0.9 and 1.1 were considered borderline and retested.

### Architect SARS-CoV-2 IgG

We used an Architect autoanalyzer (Abbott Laboratories, Inc., USA) to semiquantitatively detect anti-SARS-CoV-2 IgG. The assay is a two-step immunoassay that involves binding between the SARS-CoV-2N antigen coated on the paramagnetic microparticles and human IgG in the test sera, followed by acridinium-conjugated anti-human IgG as the secondary antibody. The chemiluminescence signals emitted were measured as relative light units (RLUs), which correlated with the amount of the specific IgG. The RLU of the sample (S) divided by the calibrator (C) yielded the index S/C ratio. Test sera with S/C ratios ≥ 1.4 were considered positive for SARS-CoV-2 IgG.

### Vitros immunodiagnostics

The Vitros Immunodiagnostics autoanalyzer (Ortho-Clinical Diagnostics, Inc., U.S.A.) is a two-step immunoassay for semiquantitatively detecting anti-SARS-CoV-2 IgG or total Ig. The use of a luminogenic substrate and an electron transfer reagent that increased the emitted light level enhanced the chemiluminescence. The S1 protein precoated on the microwells reacted with human IgG or total Ig, which subsequently reacted with either HRP-conjugated mouse anti-human IgG or anti-total Ig. The intensity of the emitted light correlated with the amount of IgG binding or the total Ig in the test sera. Values of ≥ 1 were considered positive for both SARS-CoV-2 IgG and total Ig.

### Statistical analysis

We determined the sensitivity, specificity, positive predictive value (PPV), and negative predictive value (NPV) of each serological assay using all 1005 serum samples as a whole and in certain subgroups, as appropriate. The study also determined the correlations between the index values of each assay and the NT antibody titer to assess whether the index levels correlated with the protective antibodies. We performed all analyses using the Stata program, version 16. R square (R^2^), mean, and standard deviation (SD) were analysed by GraphPad Prism version 8.4.3 for Windows (GraphPad Software, La Jolla, California, USA). The figures were drawn using GraphPad Prism version 8.4.3 for Windows (GraphPad).

## Results

### Evaluation of test performance using negative control sera

When run on the 102 COVID-19 prepandemic negative-control sera, all assays (microNT, Wantai ELISA total Ig, Architect IgG, Vitros IgG, and Vitros total Ig) yielded negative results, except one sample that was positive by the Vitros total Ig. Retesting of the one discordant result with IIF suggested that this sample was negative for SARS-CoV-2 antibodies.

### Evaluation of test performance using positive control sera

When tested on the 45 microNT antibody-positive serum samples from SARS-CoV-2-infected cases, the Wantai ELISA and Vitros total Ig detected SARS-CoV-2 antibodies in all serum samples (sensitivity = 100%) (45/45). Architect IgG and Vitros IgG had sensitivities of 88.9% (40/45) and 82.2% (37/45), respectively. We used IIF to retest the 13 problem serum samples and found that all were positive, indicating SARS-CoV-2 infection.

### Evaluation of test performance using sera of persons from the general community

Among the sera collected from the 366 individuals investigated as being at risk of SARS-CoV-2 infection in 2020, only two sera (0.55%) were true positives (Table 1), suggesting a low prevalence of infection in this population at the time of the study. Architect IgG was the only kit that did not detect either of the two positive serum samples. All other assays detected both positive specimens, showing a sensitivity of 100%. The specificity and NPV of these assays were also all above 99%. Nevertheless, based on IIF verification, each assay produced between one and three false-positive results, leading to PPVs of 40% with Wantai ELISA, 50% with Vitros IgG, 66.7% with Vitros total Ig, and 100% with microNT (Table 1).

**Table 1:** Test performance of each serological assay among persons investigated as being at risk of SARS-CoV-2 infection in the general population (N = 366)

Assays	MicroNT		Number positive IIF among discordant results	% (95% CI)			
	Pos	Neg		Sens	Spec	PPV	NPV
MicroNT	2	364		100	100	100	100
Wantai total Ig							
Pos	2	3	0 of 3	100	99.2	40.0	100
Neg	0	361		(15.8, 100)	(97.6, 99.8)	(5.3, 85.3)	(99.0, 100)
Architect IgG							
Pos	0	3	2 of 5	0	99.2	0	99.5
Neg	2	361		(0, 84.2)	(97.6, 99.8)	(0, 70.8)	(98.0, 99.9)
Vitros IgG							
Pos	2	2	0 of 2	100	99.5	50.0	100
Neg	0	362		(15.8, 100)	(98.0, 99.9)	(6.8, 93.2)	(99.0, 100)
Vitros total Ig							
Pos	2	1	0 of 1	100	99.7	66.7	100
Neg	0	363		(15.8, 100)	(98.5, 100)	(9.4, 99.2)	(99.0, 100)

Final results after IIF verification: Pos = 2, Neg = 364

*Pos* positive, *Neg* negative, *Sens* sensitivity, *Spec* specificity, *PPV* positive predictive value, *NPV* negative predictive value, *95% CI* 95% confidence interval

### Evaluation of test performance using sera of returning travellers from countries with a high prevalence of infection

Of the 492 serum samples from Thai citizens returning from foreign countries, 139 (28.3%) were positive for SARS-CoV-2 antibodies. In this higher prevalence population, the sensitivity of the assays ranged from 89.9 to 100%, and the specificities were all above 98.9%. The PPV and NPV of the assays were all over 97.2% and 96.2%, respectively, as shown in Table 2. A single serum sample tested negative by microNT, whereas it tested positive by the Wantai ELISA, Vitros total Ig, and IIF. The fluorescent-positive cells are shown in Fig. 1.

**Table 2:** Test performance of each serological assay in travellers returning from higher prevalence locations (N = 492)

Assays	MicroNT		Number positive IIF among the discordant results	% (95% CI)			
	Pos	Neg		Sens	Spec	PPV	NPV
MicroNT	138	354		99.3	100	100	99.7
Wantai total Ig							
Pos	138	5	1 of 5	100	98.9	97.2	100
Neg	0	349		(97.4, 100)	(97.1, 99.7)	(93.0, 99.2)	(99.0,100)
Architect IgG							
Pos	126	2	12 of 14	90.7	99.4	98.4	96.4
Neg	12	352		(84.5, 95.0)	(98.0, 99.9)	(94.5, 99.8)	(94.0, 98.1)
Vitros IgG							
Pos	137	4	1 of 5	98.7	98.9	97.2	99.4
Neg	1	350		(94.9, 99.8)	(97.1, 99.7)	(92.9, 99.2)	(98.0, 99.9)
Vitros total Ig							
Pos	124	2	15 of 16	89.9	99.7	99.2	96.2
Neg	14	352		(83.7, 94.4)	(98.4, 100)	(95.7, 100)	(93.7, 97.9)

Final results after IIF verification: Pos = 139, Neg = 353

### Evaluation of sera in the overall combined samples

Of the 1005 sera in the combined sample sets, 186 (18.5%) were positive for SARS-CoV-2 antibodies. The microNT and the other four commercial kits yielded discordant results in 55 serum samples, and IIF retested them. All the microNT results, either positive or negative, were supported by IIF. An exception occurred with one sample, which was positive by Wantai ELISA and Vitros total Ig but negative by microNT, resulting in a sensitivity of 99.5%, NPV of 99.9%, and specificity and PPV of 100%. The Wantai ELISA total Ig had the highest sensitivity (100%) and was followed in order by microNT (99.5%), Vitros IgG (94.6%), Vitros total Ig (92.5%), and Architect IgG (89.3%). The specificities of all commercial kits were over 99%; the PPVs and NPVs were over 96% and 97%, respectively (Table 3).

**Table 3:** Test performance of each serological assay in the overall study (N = 1005)

Assays	MicroNT		Number positive IIF among discordant results	% (95% CI)			
	Pos	Neg		Sens	Spec	PPV	NPV
MicroNT	185	820		99.5	100	100	99.9
Wantai total Ig							
Pos	185	8	1 of 8	100	99.2	96.4	100
Neg	0	812		(98.0, 100)	(98.3, 99.7)	(92.7, 98.5)	(99.6, 100)
Architect IgG							
Pos	166	5	19 of 24	89.3	99.4	97.1	97.6
Neg	19	815		(83.9, 93.3)	(98.6, 99.8)	(93.3, 99.0)	(96.3, 98.5)
Vitros IgG							
Pos	176	6	9 of 15	94.6	99.3	96.7	98.8
Neg	9	814		(90.3, 97.4)	(98.4, 99.7)	(93.0, 98.8)	(97.8, 99.4)
Vitros total Ig							
Pos	171	4	15 of 18	92.5	99.6	98.3	98.3
Neg	14	816		(87.7, 95.8)	(98.9, 99.9)	(95.1, 99.7)	(97.2, 99.1)

Final results after IIF verification: Pos = 186, Neg = 819

### Correlation between the NT antibody titers and the positive values of each assay

**Fig. 2:**
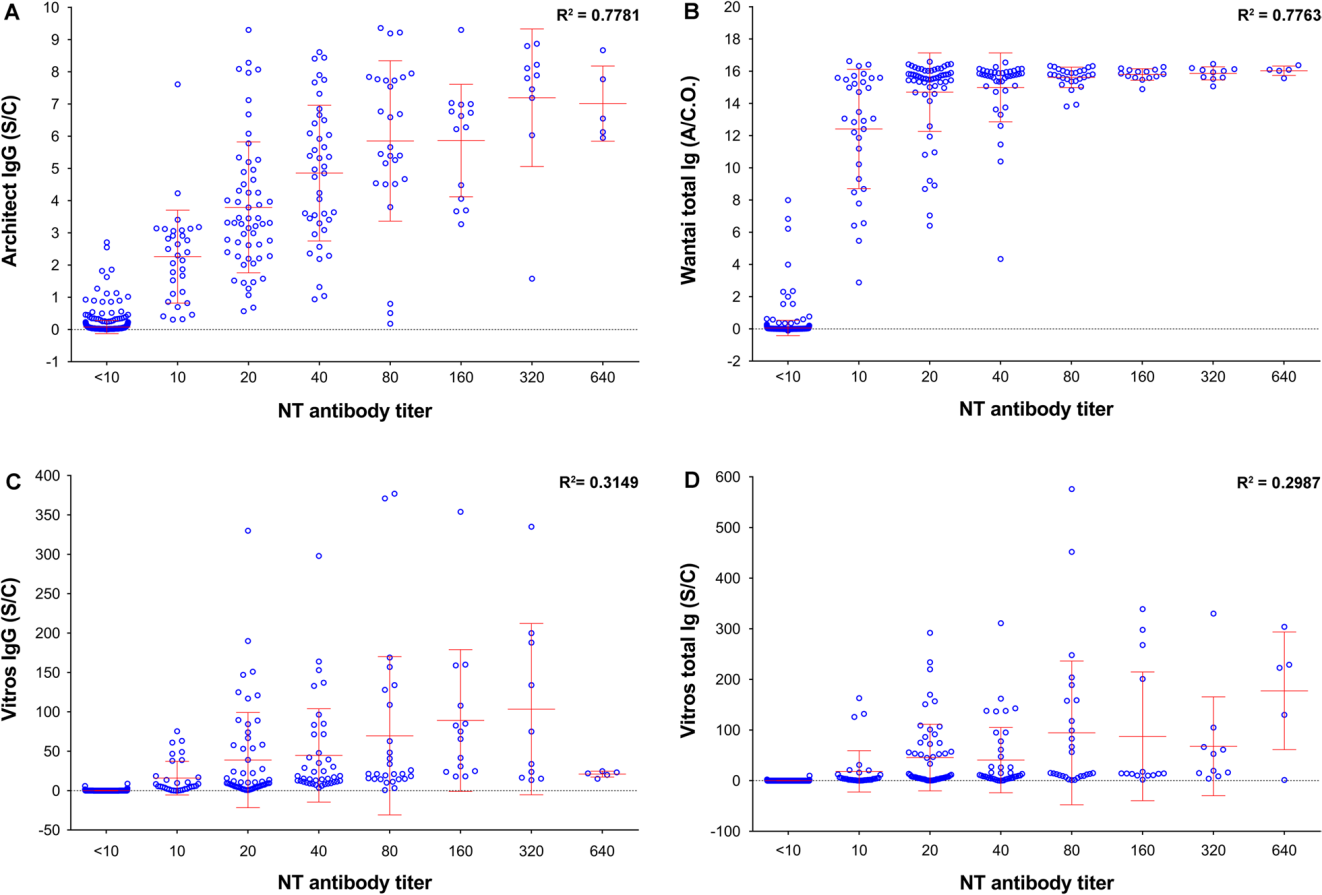
Correlation between the microNT antibody titers and the index values of each commercial assay: **A** Architect IgG; **B** Wantai ELISA total Ig assay; **C** Vitros IgG; and **D** Vitros total Ig. R square (R^2^), mean (–), and SD were analysed by GraphPad Prism version 8.4.3 for Windows (GraphPad Software, La Jolla, California, USA)

This study determined the correlations between the NT antibody titers and the index values obtained from each commercial assay. The results in Fig. 2 demonstrate a moderate degree of correlation between the microNT assay and the Architect IgG assay, which employed the N antigen (R^2^ = 0.7781), and between the microNT assay and the Wantai ELISA total Ig, which employed the RBD antigen (R^2^ = 0.7763). NT antibody titers poorly correlated with the positive values of both Vitros total Ig and Vitros IgG, which employed the S1 antigen (R^2^ = 0.2987 and 0.3149, respectively).

## Discussion

In Thailand, commercial antibody testing assays must receive approval from the Food and Drug Administration (FDA) based on having a diagnostic sensitivity of ≥ 85%, diagnostic specificity of ≥ 98%, and nonspecificity or cross-reactivity of ≤ 10%, as evaluated using a serum panel from the National Institute of Health, Department of Medical Science, MoPH. The Architect IgG, Vitros IgG, and Vitros total Ig assays have been certified for use and marketing in Thailand. The Wantai ELISA total Ig has been CE (Conformite Europeene) approved for usage in the European Union, but the company has not yet submitted a proposal for its distribution in Thailand. However, Thailand participates in the World Health Organization (WHO) population-based, age-stratified seroepidemiological UNITY study. The study aims to promote standardized epidemiological, molecular, and serological methods to facilitate international comparisons and inform decisions for the COVID-19 response [20], and the WHO offered Wantai ELISA kits to Thailand for this purpose. As a result, Mahidol University received an exemption from the Thai FDA to import these ELISA kits, allowing its inclusion in this evaluation.

The present study evaluated Wantai ELISA total Ig, Architect IgG, Vitros IgG, and Vitros total Ig test kits against microNT (supplemented with IIF verification) in detecting SARS-CoV-2 antibodies. Analysis of sera from 1005 Thai citizens showed that the four serological assays evaluated were relatively comparable in terms of sensitivity (89.3–100%), specificity (99.2–100%), PPV (96.4–100%), and NPV (97.6–100%). While all assays performed well in this evaluation, the analyses stratified by subgroups provided additional insights. As might be expected, the performance of these serological assays diminished when the prevalence of the infection was low, as in the group of 366 Thai community members, among whom only two were positive. In this group, the assays either suffered from a low sensitivity (Architect IgG) or correctly identified the positive sera but produced varying false-positive results (Wantai total Ig, Vitros total Ig, and Vitros IgG). Previous studies have also reported the reduced performance of serological assays when the infection rates were low, which significantly impacted PPV values [21–23]. According to the orthogonal testing algorithm, individuals who were initially positive by the first-line assay required retesting with a second-line assay for confirmation [24, 25]. The commercial assays used in this study did not yield false-positive results when evaluated with COVID-19 prepandemic sera (except a single serum sample investigated by Vitros total Ig).

This study sought to determine the correlation between the NT antibody titers and the index values of results from each commercial assay. The S/C ratios of Architect IgG correlated well with the NT antibody titers (R^2^ = 0.778). This correlation was unexpected because Architect IgG targets the N antigen, which contains no neutralizing epitopes. This finding suggested that both microNT and Architect IgG which target different antigenic domains, can yield comparable results on the detection of previous infection. On the other hand, the NT antibody titers also correlated well with the A/C.O. values of Wantai ELISA total Ig, which targets the RBD (R^2^ = 0.776), the primary neutralizing domain [2]. Previous investigators demonstrated that Wantai ELISA total Ig had the highest sensitivity compared to the other four commercial serological tests (BIORAD^®^ ELISA total Ig, EUROIMMUN^®^ ELISA IgG, Abbott^®^ CLIA IgG, and LIAISON^®^ CLIA IgG) when assayed in serum samples from nonhospitalized, laboratory-confirmed SARS-CoV-2 infection [26]. After 14 days postsymptom onset, Wantai ELISA total Ig had 98% agreement with microNT [22]. Other investigators also suggested that the Wantai ELISA could be a suitable substitute for the microNT assay [18]. Our study found that the NT antibody titers correlated poorly with the index values of Vitros total Ig (R^2^ = 0.2987) and Vitros IgG (R^2^ = 0.3149), which target the S1 protein. We do not have a clear explanation for this finding, as the S1 protein contains multiple antigenic sites in both the RBD and non-RBD that can induce NT antibodies [2, 27–29]. Nevertheless, chemiluminescent signals set by Vitros kits were adequate to report the positive or negative results as designed by the manufacturer. Poor correlation between the level of chemiluminescent signals and neutralizing antibodies did not affect the kit quality, because the sensitivities of both Vitros IgG and total Ig were slightly higher than Architect IgG, whereas the specificities were comparable.

In virology laboratories worldwide, microNT is the “gold standard” method for evaluating other antibody assay systems because of its high specificity. MicroNT may not always detect an early infection, as it takes time for antibodies to rise to a level that can neutralize the replicating virus at a concentration of 100 TCID50. Furthermore, some infected individuals may develop a moderate level of binding antibodies without NT antibodies. It is thus possible that serological assays that measure binding antibody activities might be more sensitive than microNT, particularly in the early phase of infection. The advantage of our study was the introduction of IIF (in which the test cells contained both N and S antigens) to verify any discordant results between microNT and a binding antibody assay. We showed that the microNT assay missed one positive sample, which the Wantai ELISA and Vitros total Ig could determine with IIF verification.

## Conclusions

Our study found that several commercial serological assays performed well for semiqualitatively determining the presence of anti-SARS-CoV-2 antibodies. However, in low prevalence populations, they may also produce false-positive results. High-throughput Architect and Vitros autoanalyzers may be the most appropriate for working on large sample sizes in countries that can afford the costs. The Wantai manual ELISA, while requiring more individual time and technical skill, may provide reliable results at a lower price. As the COVID-19 pandemic progresses, our findings can aid in selecting an appropriate serological assay for antibody detection to diagnose a past SARS-CoV-2 infection and estimate infection rates or the magnitude of the outbreaks. The assay of choice will ultimately depend on the study population, laboratory facilities, and feasibility.

## Data Availability

The datasets used and/or analyzed during the current study are available from the corresponding author on reasonable request.

## Abbreviations

microNT: Microneutralization
SARS-CoV-2: Severe acute respiratory syndrome-coronavirus-2
COVID-19: Coronavirus disease 2019
ELISA: Enzyme-linked immunosorbent assay
CLIAs: Chemiluminescence immunological assays
IIF: Indirect immunofluorescence
PRNT: Plaque reduction neutralization
RBD: Receptor-binding domain
TCID50: 50% Tissue culture infective dose
PPV: Positive predictive value
NPV: Negative predictive value
